# Analysis of the Learning Curve of Surgeons without Previous Experience in Laparoscopy to Perform Robot-Assisted Radical Prostatectomy

**DOI:** 10.1155/2018/9073807

**Published:** 2018-10-29

**Authors:** Felipe Monnerat Lott, Deborah Siqueira, Hermano Argolo, Bernardo Lindberg Nóbrega, Franz Santos Campos, Luciano Alves Favorito

**Affiliations:** ^1^Brazilian National Cancer Institute, Rio de Janeiro, Brazil; ^2^Urogenital Research Unit, State University of Rio de Janeiro (UERJ), Rio de Janeiro-RJ, Brazil

## Abstract

**Objective:**

To assess the learning curve in robot-assisted radical prostatectomy (RARP) performed by surgeons without previous experience in laparoscopic prostatectomy.

**Materials and Methods:**

We analyzed 119 patients submitted to RARP performed by two surgeons without previous experience in laparoscopic prostatectomy, with emphasis on the relevant outcomes such as continence, erectile function, and oncologic control with a minimum follow-up of 24 months. We used Fisher's exact test and the chi-square test to investigate the existence of a relationship between the variables and analysis of variance (ANOVA) to verify possible statistically significant differences between groups, at the 5% level.

**Results:**

The patients' age varied from 41 to 72 years (mean = 61.09), with 68 (57.14%) cases having intermediate or high risk. There was a consistent decline in operative time. Of the 119 patients, 80.67% were continent 6 months after surgery and 89.07% 12 months afterward, while 35.29% were potent 6 months after surgery and 60.50% 12 months following surgery. Twelve months after surgery, the trifecta outcome rate was 51.26% and the pentafecta rate was 31.09%. There was progressive postoperative improvement and maintenance of continence and sexual potency until the last patient was operated in our sample.

**Conclusions:**

Robot-assisted radical prostatectomy does not require previous experience in laparoscopic radical prostatectomy, but the learning curve is not short to achieve the plateau.

## 1. Introduction

Prostate cancer is the most common tumor other than nonmelanoma skin tumors and the second-leading cause of death due to cancer among men [1]. Robot-assisted radical prostatectomy (RARP) is well established around the world but is still in the implementation phase in various treatment centers in Brazil. Globally, the number of robotic procedures to treat prostate cancer has been growing since its introduction. In 2004, 8% of radical prostatectomies in the United States involved robot assistance [2]. This rate climbed to 53% in 2008 [2] and to 67% in 2010 [3]. Nevertheless, the learning curve of surgeons is highly variable.

To calculate the learning curve, it is necessary to have a complete or nearly complete record of the operations performed by each surgeon in his initial cases. For this reason, we collected data on 40 demographic parameters and the outcomes in our sample.

There is growing evidence that the results of various types of surgery are associated with the characteristics of the surgeon. The discussion of the surgical learning curve is extensive and controversial, with conflicting findings. The articles in general are retrospective, involving analysis only of groups of patients, with evaluation of customary postoperative data: time of hospitalization, bleeding, and time of surgery. A multicentric and prospective study compared 30 initial cases of RARP performed by surgeons with prior fellowship in robotics and by surgeons only with experience in conventional open surgery. There was a statistically significant difference in the compromised surgical margins in favor of surgeons with prior training in robot-assisted surgery (15% versus 34%, *p*=0.008), but this did not occur after the first 30 cases [4]. Another article, published in 2010, reported that the patients of inexperienced surgeons presented higher recurrence rates [5].

According to Evans et al., recurrence varies among surgeons even when they have performed a similar number of procedures [6]. The biochemical recurrence after surgical treatment of prostate cancer is a good model to analyze the association between characteristics of the surgeon and the outcomes, since adjuvant therapy is not commonly offered and recurrence is not strongly affected by other postoperative aspects.

Our hypothesis was that lack of previous experience in laparoscopic prostatectomy surgery does not interfere in the learning of RARP. To test this hypothesis, the objective of this study was to examine postoperative data to obtain the most relevant functional and oncological outcomes to construct an initial RARP learning curve, with two surgeons without prior experience in LRP, evaluating the rates of postoperative continence, erection, compromised surgical margin, and oncological control of the disease.

## 2. Patients and Methods

The experimental protocol described here was approved by our university's committee for ethical human experimentation and the ethical standards of the hospital's institutional committee on human experimentation (IRB-65099217.1.0000.5274).

We studied 133 patients who had prospectively undergone radical robotic prostatectomy to treat prostate adenocarcinoma, from June 12, 2012, to July 7, 2015, at the same institution. The patients had been submitted to RARP using the da Vinci Si system (Intuitive Surgical Inc., Sunnyvale, CA) with an average follow-up time of 45 months (varying from 27 to 64 months). It was not possible to obtain all the necessary data on 14 of those patients due to their failure to appear regularly for follow-up consultations, so they were excluded from the study. The diagnosis of cancer was done by transrectal ultrasound-guided biopsy by entities associated with the Brazilian National Health System (SUS).

All the RARP procedures were performed by two surgeons without previous LRP experience, but with more than 10 years of practice in open radical prostatectomy. The technique used was transperitoneal with bilateral opening of the endopelvic fascia.

There were no changes in the surgical technique during the study period. All the patients were first examined by fellows of our institution. The operation time was recorded by the head nurse of robotic surgery, and the data were extracted from the hospital's intranet database. The blood loss was measured at the end of each procedure, deducting the volume of physiological serum possibly administered for washing the surgical field (a bottle of physiological serum with volume markings) and also subtracting the estimated volume of urine after opening the bladder neck (estimated at 5 ml/h—by the hypotensive technique routinely used by the anesthesiology service).

The pathology analyses were performed by pathologists with extensive experience in urooncology and were in all cases reviewed by an experienced uropathologist, although according to the literature, the variation between observers regarding the surgical margin and extraprostatic extension tends to be small. [6] After this review, the extension and location of the compromised surgical margins were recorded, according to the Working Group 5 Consensus of the International Society of Urology Pathology (ISUP) [7].

To assess continence, each patient was questioned whether he used some type of the absorbent, with an additional follow-up of the pelvic physiotherapy service. If the patient had stated no use of absorbent and no contrary report was found in the physiotherapeutic records, the patient was considered continent (totally or socially continent).

The patients were considered potent if they reported successful sexual intercourse (erection degrees 3 or 4) even with the use of oral medication (phosphodiesterase type 5 inhibitor) but without intracavernous drug injection.

For statistical analysis of the learning curve, the patients were divided into four groups (according to the year of surgery), where group 1 contained patients operated in 2012, group 2 in 2013, group 3 in 2014, and group 4 in 2015.

## 3. Statistical Analysis

We used Fisher's exact text and the chi-square test to investigate the existence of a relation between the variables and analysis of variance (ANOVA) to verify a statistically significant difference between the groups, at the 5% level. For further analysis, the variables that were significant in the ANOVA were investigated by the Tukey test.

## 4. Results

The age of our patients varied from 41 to 72 years (mean= 61.09), with 68 (57.14%) being cases of intermediate or high risk. The surgery lasted for an average of 238.49 minutes (average console time of 189.28 minutes), and the average hospital stay was 2.43 days. The mean PSA level was 8.23 ng/ml (ranging from 1 to 23 ng/ml). The means and medians of the prostate weight, BMI, and postoperative bleeding and the comparisons of the means can be seen in Table 1.

In 25.21% of the cases, we observed extraprostatic extension, and in 38.65%, we noted compromised surgical margin. Of these compromised margins, 42.55% were unique, and of these, 75% were focal (<3 mm), and 69.38% were radially located. In the anatomopathological review, one case was downgraded and one was upgraded regarding the pathological staging, and there were four alterations in the report from compromised surgical margin (SM+) to free surgical margin (SM−), and vice versa. We observed 4.54% pT3 rate in group 1, 23.07% in group 2, 20% in group 3, and 39.13% in group 4.

Of the 119 patients, 80.67% were continent 6 months after surgery and 89.07% after 12 months. With respect to sexual potency, 15% of the patients had no interest in engaging in sex before the surgery. We observed that 35.29% were potent 6 months after surgery and 60.50% after 12 months. Twelve months after surgery, 51.26% of the outcomes were trifecta and 31.09% were pentafecta.

Regarding complications, 7.56% of the patients presented mild complications (Clavien I-II) and 4.20% suffered more severe ones (Clavien III-IV). In 29.41% of the cases, there was persistence of the disease, high or increasing PSA associated with compromised surgical margin, or biochemical recurrence 24 months after surgery.

Of the 119 patients, 52 were operated by surgeon A and 67 by surgeon B. There was no significant difference between the variables studied when comparing the two surgeons, confirming that their patients had the same profile regarding quantitative variables.

We observed that HAS had a statistically significant relation with erectile function two years after surgery. The majority of the patients did not have HAS and mainly had erection degree of 3. For the other variables, “seeking sexual relations?” and “trifecta 12 m,” the *p* values were lower than 0.05, indicating the existence of a relation between the variables.

The ANOVA presented a *p* value lower than 0.05 for the variables PSAD, follow-up time, bleeding, console time, and surgery time. Of particular note was the reduction of surgery time between the years: it was 64% shorter in group 4 (most recent) than in group 1.

**Figure 1:**
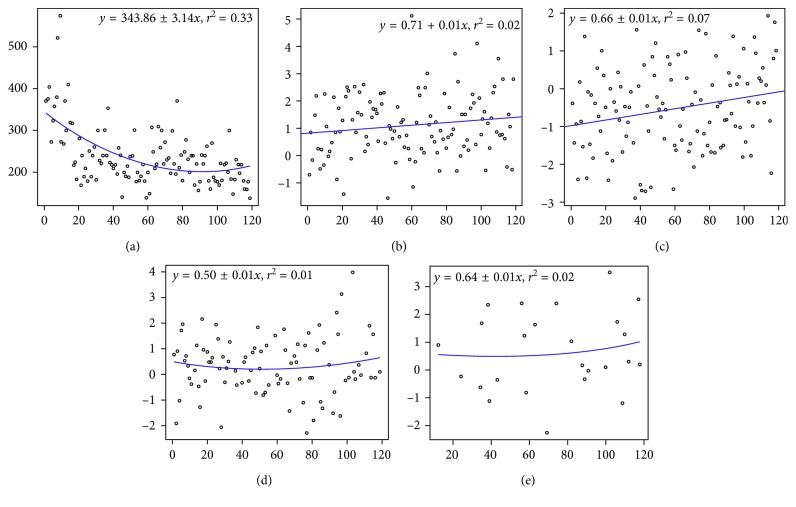
The figure shows the linear regressions of the learning curve along the series regarding the outcomes surgery time, urinary continence, and sexual potency 12 months after surgery, along with the multiple surgical margin rates. (a) Surgery time (in minutes–*y* axis) with progression of cases (number–*x* axis). The arrow marks the start of the performance of obturator lymphadenectomy, followed later by extended lymphadenectomy; *R*^2^ = 0.33; (b) continence 12 months after surgery; the best results approach line 1, *R*^2^ = 0.06; (c) erection 12 months after surgery; the best results approach line 1; *R*^2^ = 0.02; (d) compromised surgical margins in pT2 cases; the best results approach line 1; *R*^2^ = 0.01; (e) compromised surgical margins in pT3 cases; the best results approach line 1; *R*^2^ = 0.02.

The number of minutes at the console also declined markedly with the passage of years. The lowest was in group 4, operated in 2015, with *p* < 0.0001, compared with the surgeries performed in 2012, 2013, and 2014, even with worse PSA density in the cases of surgery in 2014 compared to the cases operated in 2012 and 2013 (*p*=0.01 and 0.03, respectively). Average bleeding was lowest in group 2, and the reductions between groups 3 and 2 (*p*=0.009) and between groups 4 and 3 (*p*=0.03) were most notable. In our sample, the patients were operated when presenting higher PSA levels in relation to prostate volume, and even with this adverse prognostic factor, the operative factors progressively improved.  Figure 1 shows the learning curve along the series regarding the outcomes surgery time, urinary continence, and sexual potency 12 months after surgery, along with the multiple surgical margin rates in pT2 and pT3 cases along with the multiple surgical margin rates.

The *R*^2^ values of the learning curve were 0.33 for surgical time, 0.02 for continence 12 months after surgery, 0.06 for erection, 0.01 for compromised surgical margin in pT2 and 0.02 for compromised surgical margin in pT3 after that time lapse, and 0.07 for compromised surgical margins. The best value was quantified as 1. In other words, continent = 1, level 4 erection = 1, and so on for all the variables. Therefore, the nearer the lines are to 1, the better the performance was for that variable. The graphs show a progressive improvement and maintenance of postoperative continence and sexual potency until the last patient was operated in our sample. This indicates that the plateau to be reached, after which there is no further significant improvement in the postoperative outcomes, was not attained. The same happened about the surgical margins. The results were better in pt3 than in pT2 cases along the cases done. This was expected due to the low number of cases operated by each surgeon.

For the surgical margin, we attributed a score of “0” to no compromise, so the trend line is inclined and near 0, indicating the margin tended not to be compromised as the surgeon gained experience.

## 5. Discussion

Postoperative morbidly and mortality are not good parameters to analyze the learning curve because they can be influenced not only by the surgeon's experience but also by the skill of the anesthesiologist and the training and experience of the postoperative intensive care team. A previous study found that the learning curve for LRP to reach a plateau was about 250 cases, with little improvement regarding recurrence after the surgeon had reached 250 procedures [8]. The biochemical recurrence rate after five years was 17.9% (surgeons with 10 cases) versus 10.7% (surgeons with more than 250 cases). When comparing surgeons with fewer than 10 cases against those with more than 250 cases, there was a reduction in the relative risk of 7.2%, meaning that for each 14 patients operated by the surgeon with less experience in comparison with the same measure for the more experienced surgeon, one patient will present recurrence.

In another study working with the same database as the one described above, but instead of correlating SM with BCR, the authors found a SM+ rate of 36% for surgeons with fewer than 50 cases versus 11% for surgeons with more than 1,000 cases (*p*=0.017) [9]. Despite the natural assumption that the surgeon with fewer compromised SM outcomes would have lower BCR, the data from that study indicated independence of these variables. There was a correlation between SM+ and BCR HR of 2.1 (*p* < 0.0005) [10], but this association is weak. A SM+ has 1/4 of the effect of a primary Gleason grade, 1/3 of the effect of a seminal vesicle invasion, and 50% of the effect of an extraprostatic extension to BCR. Obviously, SM is important, but it is questionable to employ it to assess the change in the surgical technique or as feedback to surgeons.

Vickers and colleagues, in a multicentric and retrospective cohort study of 4,702 patients, adopting recurrence criteria of PSA > 0.2 ng/ml (4 institutions) and PSA > 0.1 ng/ml (3 institutions), found that these recurrence criteria did not affect the probability of the same outcome for a surgeon individually. They also found the learning curve to be longer for pure laparoscopic surgery was compared to the open technique, and the results improved up to 750 procedures, when they reached a plateau. When the surgeon already had experience in open surgery, the results were better [11].

Another important point to consider is the fact that few surgeons manage to perform a large enough number of operations to reach a proficiency plateau. More than 80% of American surgeons performed fewer than 10 radical prostatectomies in 2005. Considering that the working life of a surgeon is around 25–30 years, it is estimated that the large majority do not reach this minimum of 250 operations. This means that the majority of patients receive treatment from surgeons that are not experienced enough to have fully hone their skills. Only around 4% of surgeons would reach the plateau in 10 years. In New York City, a metropolis with several quaternary hospitals, 84% of the surgeons in 2005 performed fewer than 10 radical prostatectomies, with only 3.4% performing 50 or more [12].

Another study, with 400 patients submitted to RARP between 2012 and 2015 by a single surgeon with experience of 600 cases of LRP, found an average surgery time of 187.2 minutes, with an estimated blood loss of 240.9 ml. The SM+ rate was 20% (64.6% at pT3 and 35.4% at pT2). With respect to location, 52.4% of the SM+ cases were apical, while we found that the majority of the compromised surgical margins were radial, perhaps due to an exaggerated attempt to achieve good neural preservation. In that other study, there was no significant difference in the SM+ rate as the number of cases progressed, possibly because the surgeon already had good experience in LRP [13].

Machado and colleagues [14] reported that surgeons with more than 200 LRP cases obtained similar oncological results in the first 60 cases using RARP. They found mean operating time of 236 minutes and blood loss of 245.6 ml. They also observed a complication rate of 6.6%, pT2 of 81.6%, pT3 of 18.4%, continence after 6 months of 93.3%, potency after 6 months of 70%, and SM+ rate of 21.6%, with the complications of RARP varying from 1.5% to 17.8%. We observed similar values for surgery time, blood loss, and complications, even though the surgeons had no previous experience in laparoscopic radical prostatectomy.

A recent study compared the results of a surgeon experienced in the use of robot assistance (with analysis after 70 procedures) against those of a surgeon only with experience in open radical prostatectomy [15]. The result reported for operation time was similar to that found in our sample (238  min), and the SM+ rate was 24% in the patients operated with the da Vinci platform.

A cohort study with prospective data collection on 500 patients operated between 2005 and 2012 (RARP) revealed a significant decline in operation time and bleeding rates between group I (1–250 cases) and group II (251–500 cases), even though group II had more advanced cases. There was no significant difference regarding SM+ rates, even though there was a decrease from 38.4% to 30.0% between the two groups (*p*=0.059). There was a reduction from 49% (group I) to 32.6% (group II) in the SM+ rate at pT3 (*p*=0.007) [16]. We did not obtain similar reduction in the rates of compromised surgical margin, probably because of the smaller sample.

A meta-analysis comparing RARP with LRP [17] found the following outcome values for the latter procedure: continence at 12 months of 91.5%, potency at 12 months of 81% (results similar to ours), SM+ of 14.4% in a sample with 44% rate of extraprostatic disease, and a biochemical recurrence rate of 9.8%. The authors stated that various factors might have influenced the postoperative continence (patient's age, prostate weight, BMI, nerve sparing, preservation of the bladder neck, sparing of the pubovesical complex, reconstruction/preservation of the anatomical structures, urethral length, and comorbidities) [18].

An analysis of cases initially operated by four surgeons showed that the learning curve to obtain postoperative continence did not reach a plateau even after 200 cases [19], which would also explain what happened in our cases.

Another retrospective study analyzed the learning curve with a total sample of 500 patients operated by two surgeons, where the patients were divided into sequential groups of 25 patients. The authors found a statistically significant reduction in operation time, blood loss, and hospital stay at each 25 procedures. There was a significant decrease for one surgeon and a declining trend for the second one regarding postoperative complications. There was no difference regarding the rates of compromised surgical margins between the groups of patients [20].

The rates of SM+ in robot-assisted radical prostatectomy are variable in the literature, from 6.5 to 32% in general, as described by Yossepowitch [21] and Novarra and colleagues [22], with an average rate of 15% or up to 36% for surgeons with an experience of fewer than 50 cases and 16.7% for surgeons with a background of 51 to 140 cases. In turn, Atug et al. [23] reported that a surgeon's skill increases after the first 30 cases, with rates of compromised surgical margin falling from 45.4% to between 21.2 and 11.7%. Our findings for compromised SM rates are therefore within the parameters found previously in the literature. The total positive surgical margin rate in our sample was 38,65% (34,8% of the PSM occurred in pT3 and 65,2% in pT2). In contrast, Patel and colleagues [24] reported lower SM+ rates: 12.2% for from 1 to 300 cases operated, 6.6% for 301–600 cases, 13.6% for 601–900 cases, 11% for 901–1,200 cases, and 1.8% with an experience of 1201–1500 procedures.

Another retrospective study (2002–2012) in a hospital in the United Kingdom analyzed 592 minimally invasive radical prostatectomies. The SM+ rate was 30.6%, with demographic data and follow-up times similar to those of our sample. The authors found BCR in 10.7% of the cases with SM+ and in 5.1% of the cases with SM−, with an average follow-up time of 30.3 months [25]. An analysis of the initial series of the first 100 cases of three surgeons over a period of nine years [26] observed an increase in the number of pT3 cases in the second group of 50 cases operated, with only a small increase in the occurrence of SM+, similar to our findings.

Finally, Thompson and colleagues [27] conducted a comparative analysis of the initial cases of robot-assisted radical prostatectomy with the cases of open radical prostatectomy of the same surgeon, who had performed more than 3,000 open surgeries and more than 2,000 robot-assisted operations. The results of sexual potency, continence, and compromised surgical margin were significantly better in the cases of open surgery compared to the initial robotic ones, which was reverted soon thereafter. The plateau of the RARP learning curve was 330 cases for sexual potency, 365 for early return of continence, 659 for complete resolution of incontinence, 484 for reduction of the compromised surgical margin rate, and 226 cases for biochemical recurrence.

We should mention the most important weak points of this study: the very small sample, the short follow-up interval for a secure analysis of biochemical recurrence and compromised surgical margin, the lack of analysis based on quality of life questionnaires, and the fact that some patients were submitted to complementary radiotherapy before the PSA exceeded the barrier of 0.2 ng/ml (a traditional threshold considered for biochemical recurrence).

## 6. Conclusion

Our results and their comparison with other reports in the literature demonstrate that the learning curve for robot-assisted radical prostatectomy is extended, not reaching a plateau with fewer than 100 surgery cases, despite the progressive improvement during the course of the series and the good general results.

In light of the very long learning curve, we recommend regionalization of prostate cancer treatment because surgeons with a larger number of cases under their belts reduce the hospital expenses by lowering the rates of complications and costs for adjuvant treatments.

## Figures and Tables

**Table 1:** The table shows the descriptive analysis and comparison of the means of the 4 groups of patients submitted to robotic radical prostatectomy. Group 1 contained patients operated in 2012, group 2 in 2013, group 3 in 2014, and group 4 in 2015.

Variable	X	Median	SD	CV	Minimum	Maximum	X of group 1	X of group 2	X of group 3	X of group 4	*p* value
Age (years)	61.09	62.00	5.71	9.34	41.00	72.00	61.28	59.62	61.89	62.30	0.2290
Body mass index	24.8	25	2.68	10.8	18.09	33.00	21.23	24.11	25.75	19.30	0.6570
Prostate weight (g)	29.71	31.00	11.70	39.40	7.00	52.00	27.41	29.31	31.03	30.57	0.6960
PSA density (%)	24.80	25.00	2.69	10.84	18.00	33.00	23.55	24.11	25.75	25.71	0.0017
Probability of extraprostatic extension (PARTIN) (%)	20.29	22.00	4.39	21.63	9.00	25.00	21.23	20.31	20.34	19.30	0.5440
Follow-up (months)	45.30	46.00	10.92	24.10	27.00	64.00	60.68	51.15	39.06	30.17	0.0001
Hospitalization time (days)	2.44	2.00	2.25	92.32	1.00	21.00	3.09	2.23	2.09	2.70	0.3450
Bleeding (milliliters)	207.48	150.00	174.34	84.03	10.00	1200.00	245.00	153.33	277.71	156.52	0.0049
Console time (minutes)	189.29	180.00	63.14	33.36	100.00	470.00	265.23	177.56	178.86	152.39	0.0001
Surgery time (minutes)	238.50	220.00	71.59	30.02	140.00	570.00	324.59	218.08	234.29	197.17	0.0001

X = mean; CV = coefficient of variation; SD = standard deviation.

## Data Availability

The Excel prospective collected data used to support the findings of this study are available from the corresponding author upon request.

## Abbreviations

ASA: American Association and Anesthesiology (classification of the physical state)
BCR: Biochemical recurrence
BMI: Body mass index
CLAVIEN: Clavien–Dindo classification of surgical complications
cT: Clinical staging
HAS: Systemic arterial hypertension
INCA: National Cancer Institute
LRP: Laparoscopic radical prostatectomy
ORP: Open radical prostatectomy
Pentafecta: Oncological control of the disease + urinary continence + sexual potency + negative surgical margin + absence of postoperative complications
PSAD: PSA density
pT: Pathological staging
RARP: Robot-assisted radical prostatectomy
SHIM: Sexual Health Inventory for Men
SM+: Compromised surgical margin
Trifecta: Oncological control of the disease + urinary continence + sexual potency
USA: United States of America
XRT: X-ray therapy.
